# Supplementary material for: Targeted therapies of inflammatory diseases with intracellularly gelated macrophages in mice and rats
Source: Nat Commun. 2024 Jan 6;15:328. doi: 10.1038/s41467-023-44662-5 (PMC10771422; doi:10.1038/s41467-023-44662-5)
Supplement: Supplementary file 1 — Supplementary Information [file 41467_2023_44662_MOESM1_ESM.pdf]

Supplementary Information for

Targeted therapies of inflammatory diseases with intracellularly gelated macrophages

Cheng Gao,[1,2,+] Qingfu Wang,[1,+] Yuanfu Ding,[1,3,+] Cheryl H.T. Kwong,[1] Jinwei Liu,[1] Beibei Xie,[1] Jianwen Wei,[1] Simon M. Y. Lee,[1,2] Greta S. P. Mok,[2,3] Ruibing Wang[1,2,]*

Affiliations:

[1] State Key Laboratory of Quality Research in Chinese Medicine, Institute of Chinese Medical Sciences, University of Macau, Taipa, Macao 999078, China.

[2] MoE Frontiers Science Center for Precision Oncology, University of Macau, Taipa, Macao 999078, China.

[3] Biomedical Imaging Laboratory (BIG), Department of Electrical and Computer Engineering, University of Macau, Taipa, Macao 999078, China.

* Correspondence should be addressed to Prof. Ruibing Wang at rwang@um.edu.mo

[+] These three authors contributed equally to this work.

Supplementary Figures

Supplementary Fig. 1. Preparation of Phe-CS.
Supplementary Fig. 2. Characterization of Phe-CS based hydrogel.
Supplementary Fig. 3. Membrane lipid order of GMs.
Supplementary Fig. 4. Cytokine neutralization efficiency of GMs, MM-NPs, GRBCs and EGMs.
Supplementary Fig. 5. Photographs of the rat paws taken at all time points in different treatment groups during therapeutic treatment.
Supplementary Fig. 6. Therapeutic efficacy of hydrogel, MAs, DS+aTNF-α and GC in CIA rats.
Supplementary Fig. 7. Photographs of the rat paws taken at all time points in different treatment groups during prophylactic treatment.
Supplementary Fig. 8. The damage evaluation on CIA rats after phylactic treatment with different formulations.
Supplementary Fig. 9. Ex vivo biodistribution and in vivo pharmacokinetics of GMs in AP mice.
Supplementary Fig. 10. Gating strategy of flow cytometry analysis on the filtration of macrophages ($F4/80^{+}CD11b^{+}$ cells) and proinflammation polarized macrophages (the ratio of $CD86^{+}$ Cells, $CD40^{+}$ cells and $CD80^{+}$ cells in $F4/80^{+}CD11b^{+}$ cells) in lung tissues.
Supplementary Fig. 11. Therapeutic efficacy of hydrogel, MAs and GRBCs on acute lung inflammation in AP mice.
Supplementary Fig. 12. Fluorescence imaging on the filtration of neutrophils ($CD11b^{+}Gr\text{-}1^{+}$ cells) in the lungs of treated mice, and semi-quantitative analysis by ImageJ software.
Supplementary Fig. 13. In vivo safety evaluation of GMs on SD rat.
Supplementary Fig. 14. Evaluation of autoimmune response in liver from GMs treated mice.

Supplementary Tables

Supplementary Table 1. Dilutions and catalogue numbers of all used antibodies.

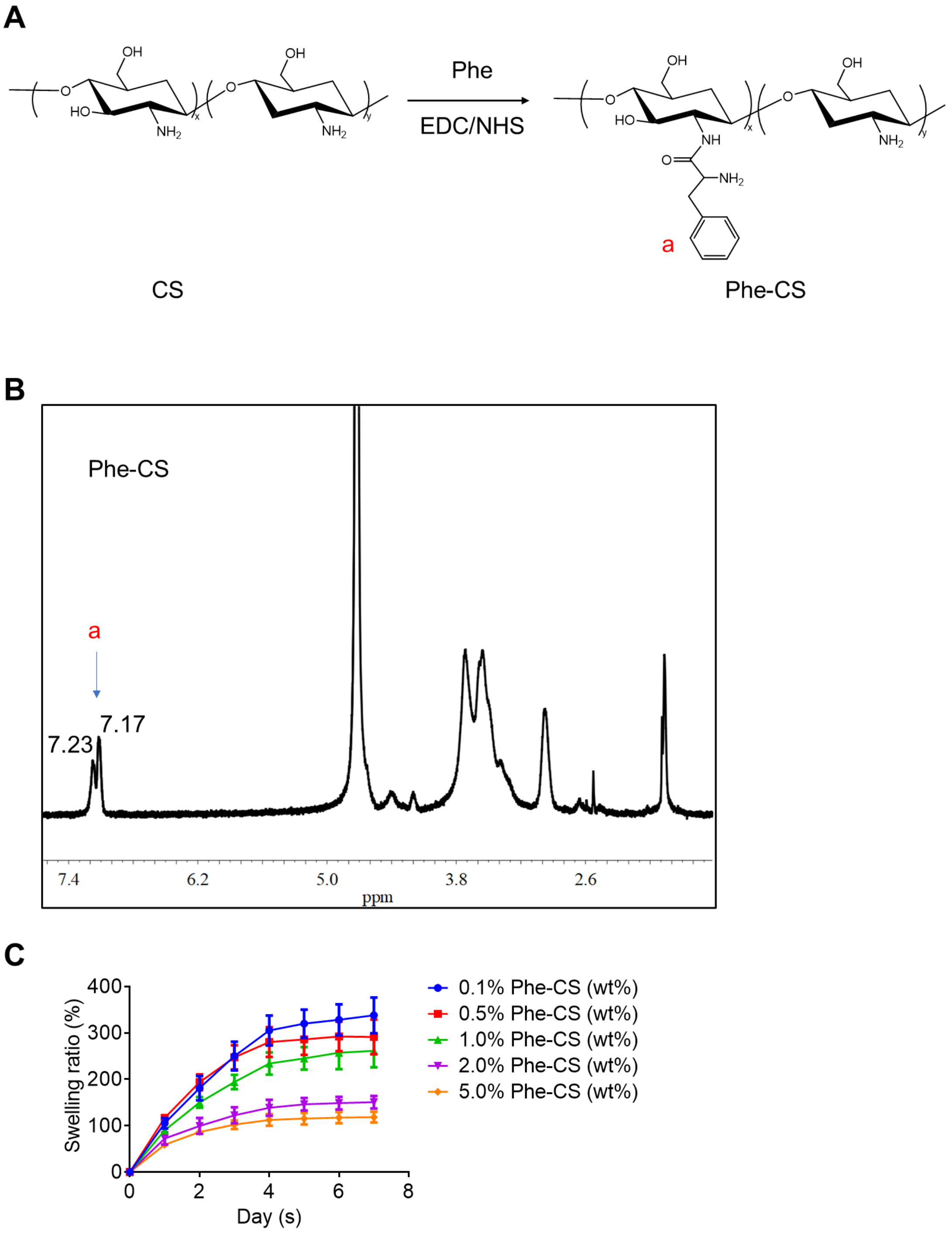

Supplementary Fig. 1. Preparation of Phe-CS. (**A**) Synthetic process of Phe-CS. (**B**) ^{1}H NMR spectrum of Phe-CS. (**C**) Swelling profiles of hydrogels prepared from different concentrations of Phe-CS (0.1%, 0.5%, 1%, 2% and 5%, wt%) and 50 μM of CB[8] in PBS solution at 37 °C. All data was presented as mean ± s.d. (n = 3).

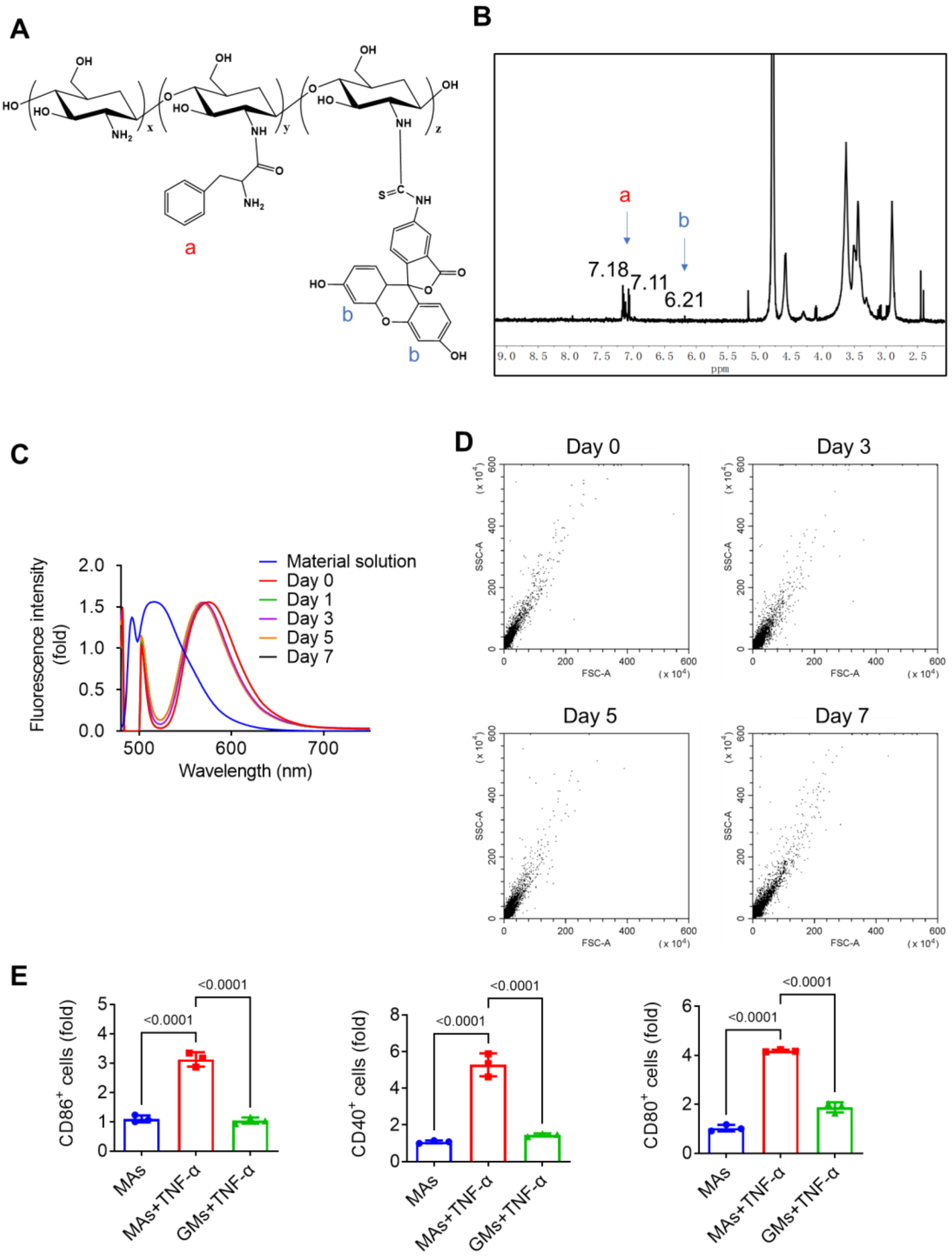

Supplementary Fig. 2. Characterization of Phe-CS based hydrogel. (**A**) Chemical structure of FITC conjugated Phe-CS. (**B**) ^{1}H NMR spectrum of FITC conjugated Phe-CS. (**C**) Phe-CS was conjugated with FITC to label hydrogel for fluorescence correlation spectroscopy (FCS) analysis. After storage in serum for different durations (0, 1, 3, 5 7 days), the fluorescence emission spectrum of hydrogel was determined at an excitation wavelength of 480 nm. (**D**) Plot scattering of GMs after storage in serum for 7 days, analyzed by flow cytometry. (**E**) Quantitative results of the proinflammatory polarization of MAs and GMs ($CD86^+$ cell, $CD40^+$ cell and $CD80^+$ cell) after treatment with 10 μM of TNF-α for 12 h. All data was presented as mean ± s.d. (n = 3). All statistical analyses were conducted using One-Way ANOVA.

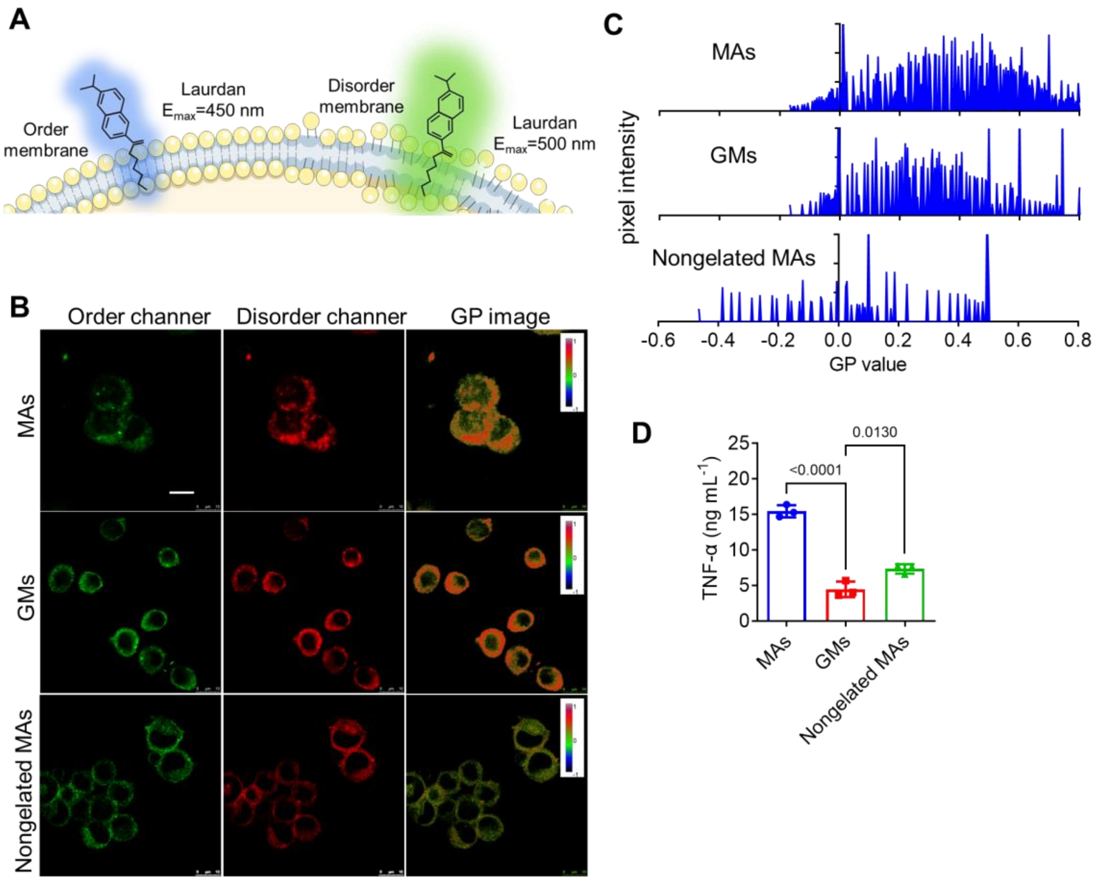

Supplementary Fig. 3. Membrane lipid order of GMs. (**A**) Schematic illustration of evaluation on membrane lipid order by using laurdan staining. (**B**) Representative pseudo-colored GP-intensity-merged images of order channer and disorder channer in laurdan stained MAs, GMs and Nongelated MAs, respectively. Ordered membrane domains were shown in orange. Scale bar: 10 μm. (**C**) Histograms of GP values comparing the membrane lipid order of MAs, GMs and Nongelated MAs. (**D**) MAs, GMs and Nongelated MAs at a number of $5x10^6$ were incubated with 10 μg mL^{-1} of TNF-α for 12 h, and the supernatant TNF-α concentration was determined by Elisa kit. Representative photos in (B) came from three independent experiment on three different cell samples (n=3). Data in (D) was presented as mean ± s.d. (n=3), and statistical analysis was conducted using One-Way ANOVA.

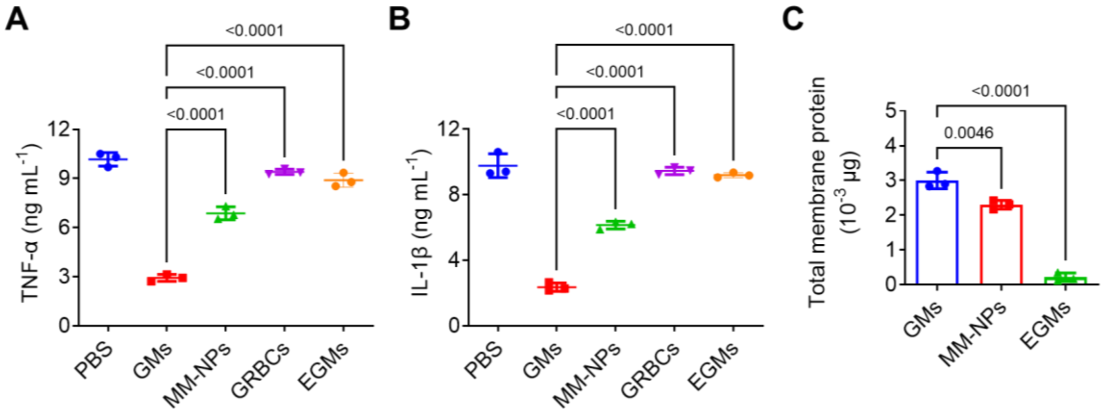

Supplementary Fig. 4. Cytokine neutralization efficiency of GMs, MM-NPs, GRBCs and EGMs. (**A** and **B**) GMs, MM-NPs and EGMs prepared from same number of macrophages ($3x10^6$), and $3x10^6$ of GRBCs were incubated in 1 mL of PBS solution containing 10 ng mL^{-1} of TNF-α (A) and 10 ng mL^{-1} of IL-1β (B) for 1 h, respectively. After removing the precipitates, the supernatant cytokine concentrations were measured by Elisa kits. (**C**) Membrane protein contents of GMs, MM-NPs and EGMs prepared from same number of macrophages ($3x10^6$) were analyzed by BCA protein assay. All data was presented as mean ± s.d. (n = 3). All statistical analyses were conducted using One-Way ANOVA.

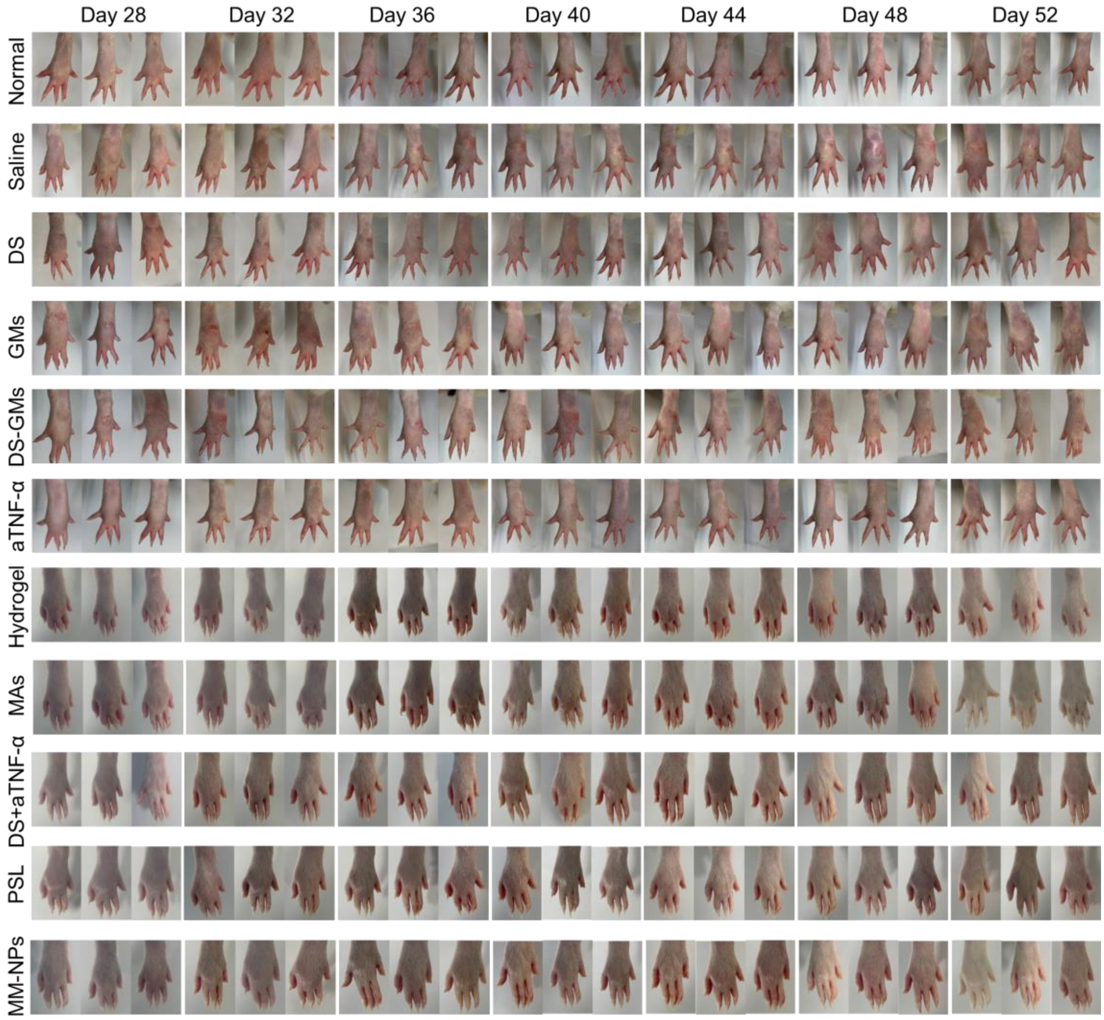

Supplementary Fig. 5. Photographs of the rat paws taken at all time points in different treatment groups during therapeutic treatment. The swelling paws of CIA rats after 4 weeks of arthritis development were injected with saline, DS, GMs, DS-GMs, aTNF-α, and DS+ aTNF-α at $3x10^6$ of GMs per rat, 1 mg kg^{-1} of DS and 1 mg kg^{-1} of aTNF-α (n=3). Rats treated with 2 mg kg^{-1} of hydrogel, $3x10^6$ of MAs and 2 mg kg^{-1} of PSL, and MM-NPs prepared from $3x10^6$ of MAs served as control groups. After treatment once every four days until Day 52, photos at pre-determined time points demonstrated the progression of mouse paw inflammation.

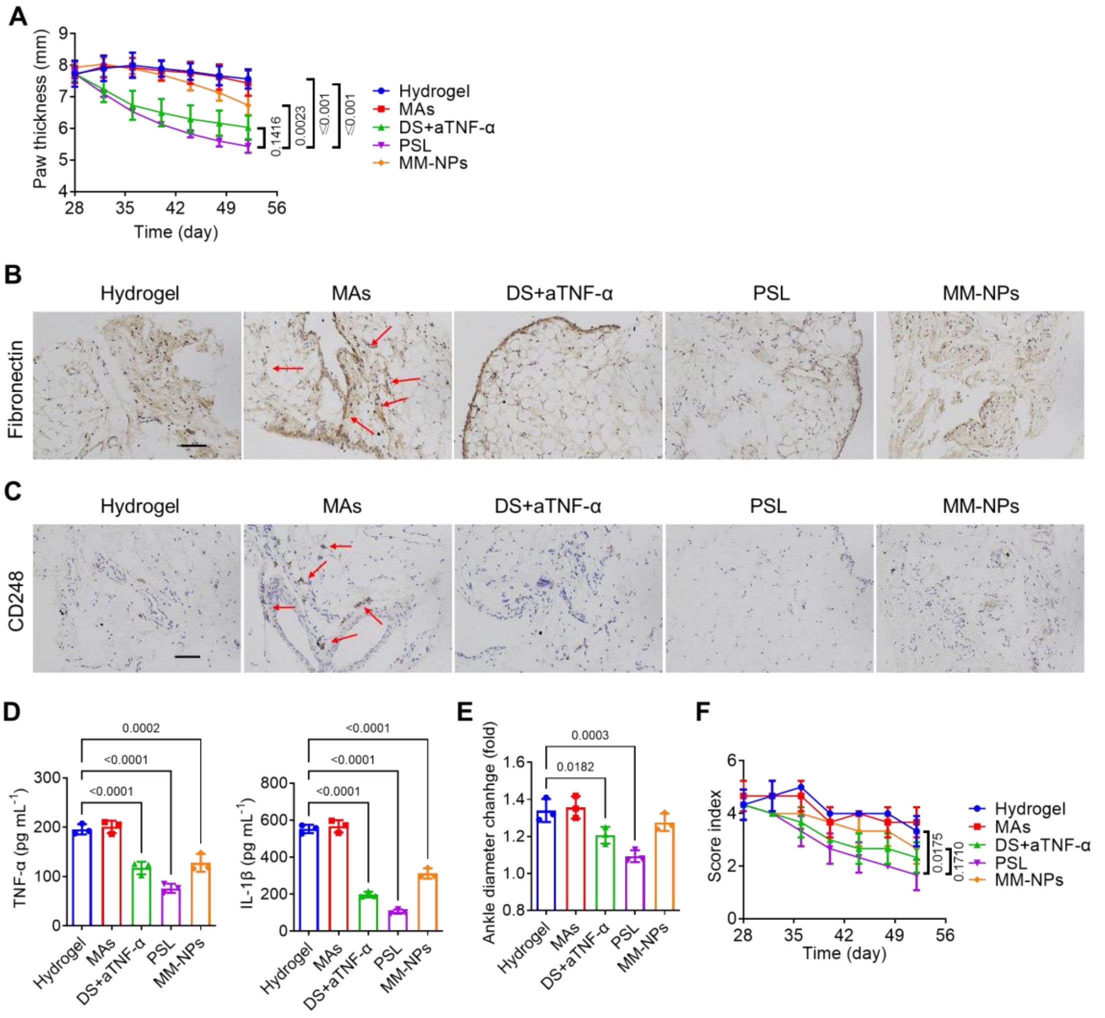

Supplementary Fig. 6. Therapeutic efficacy of hydrogel, MAs, DS+aTNF-α and GC in CIA rats. (**A**) The changes of paw thickness in CIA rats after treatments with DS+ aTNF-α at 1 mg kg^{-1} of DS and 1 mg kg^{-1} of aTNF-α, 2 mg kg^{-1} of hydrogel, $3x10^6$ of MAs, MM-NPs prepared from $3x10^6$ of MAs, and 2 mg kg^{-1} of PSL. (**B** and **C**) Representative images of Fibronectin staining (B) and CD248 staining (C) on the on the synovial intimal lining from treated rats. Scale bar: 100 μm. (**D**) The concentrations of serum TNF-α and IL-1β at the endpoint of treatment. (**E**) The ankle diameter changes of treated rats compared to that of the normal rats recorded at Day 52. (**F**) The overall arthritis score of the treated rats recorded every four days with a total of 28 days after arthritis induction. Representative photos in (B) and (C) came from three independent experiment on three different synovial intimal linings (n=3). All data was presented as mean ± s.d. (n = 3). All statistical analyses were conducted by using One-Way ANOVA.

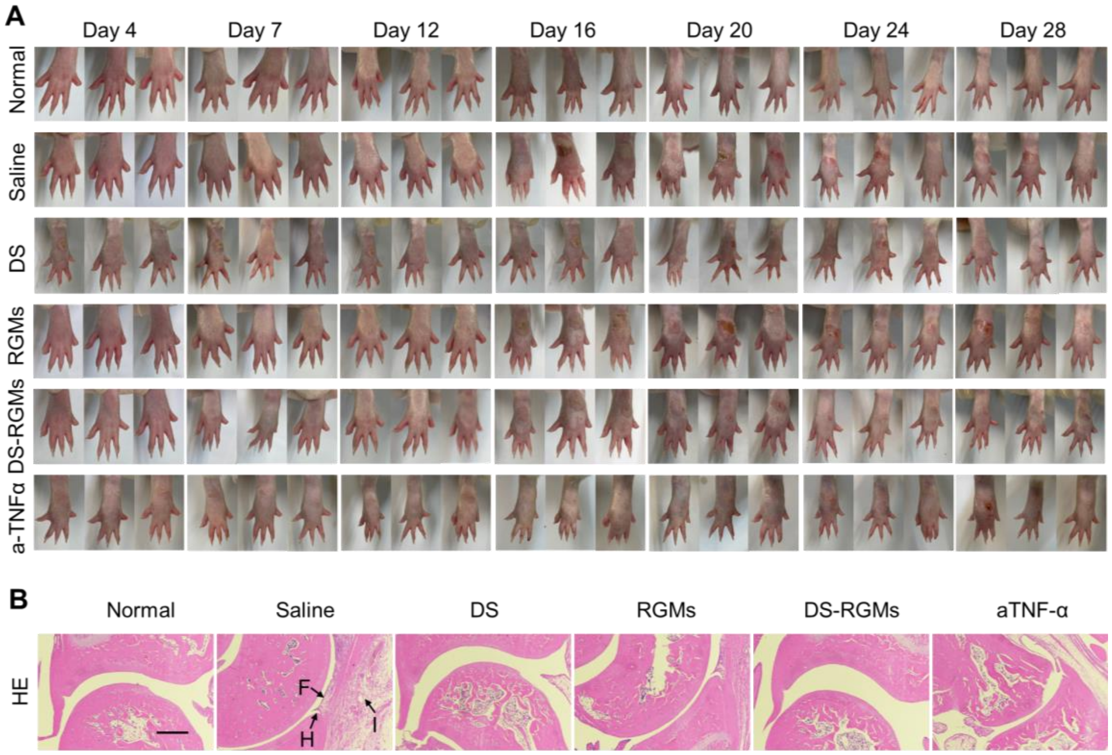

Supplementary Fig. 7. Photographs of the rat paws taken at all time points in different treatment groups during prophylactic treatment. (**A**) CIA rat with early-stage arthritis was in situ administered with saline, DS, RGMs and DS-RGMs at $3x10^6$ of GMs per rat and 1 mg kg^{-1} of DS once every four days until Day 28 (n=3). 1 mg kg^{-1} of aTNF-α treated rat served as control groups. Representative photos at determined time points demonstrated the progression of mouse paw inflammation. (**B**) HE staining on ankle sections from treated rats. F: synovial membrane fibrillation. H: synovium hyperplasia. I: immune cell infiltration. Scale bar: 200 μm. Representative photos in (B) came from three independent experiment on three different ankle sections (n=3).

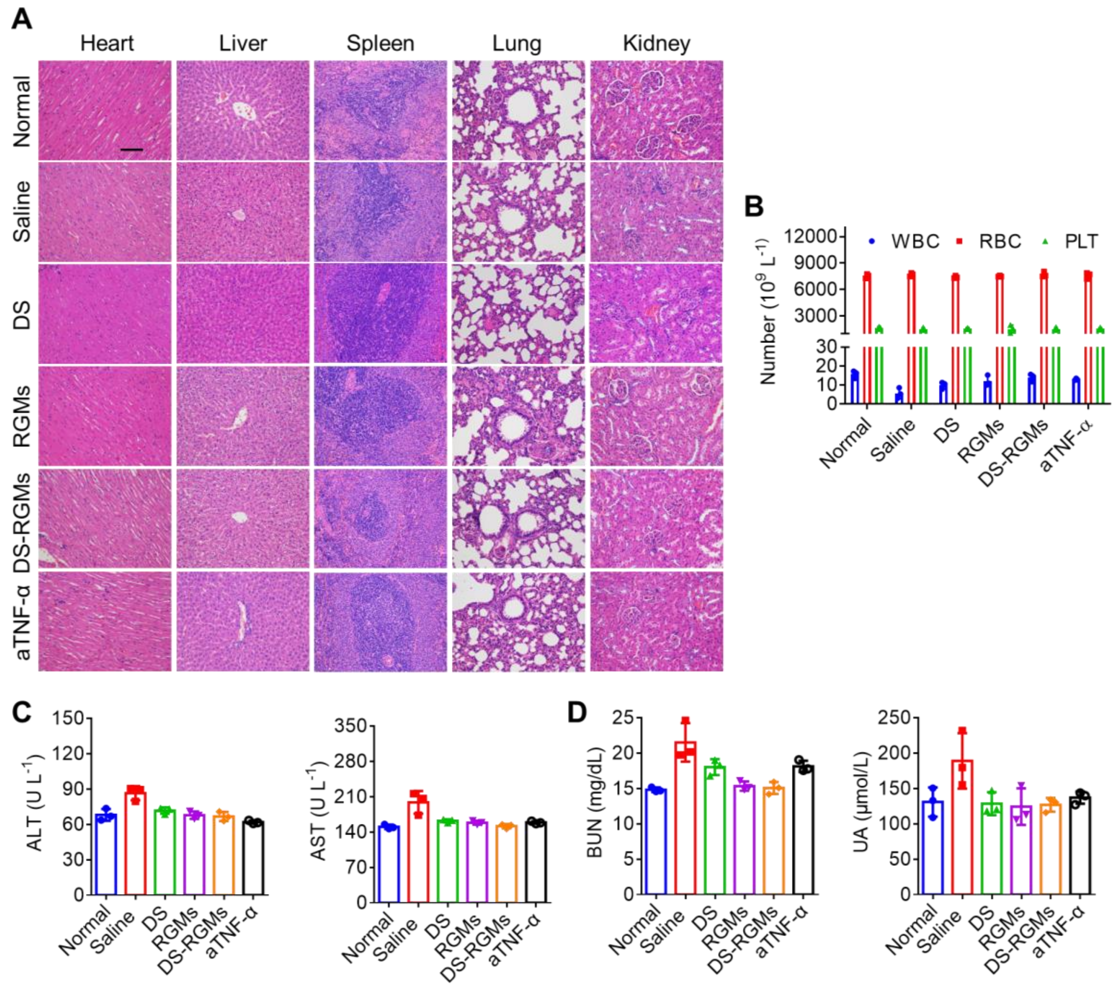

Supplementary Fig. 8. The damage evaluation on CIA rats after phylactic treatment with different formulations. (**A**) At endpoint of phylactic treatment, different organs (heart, liver, spleen, lung and kidney) and bloods were collected from treated mice (n=3). HE staining was conducted in the heart, liver, spleen, lung, and kidney. Scale bar: 100 μm. (**B**) The number of WBCs in blood was measured by hemocytometer. (**C** and **D**) The level of ALT, AST (C), BUN and UA (D) in the serum were analyzed. The experiments were repeated for three times (n = 3) and data was presented as mean ± s.d. All statistical analyses were conducted using One-Way ANOVA.

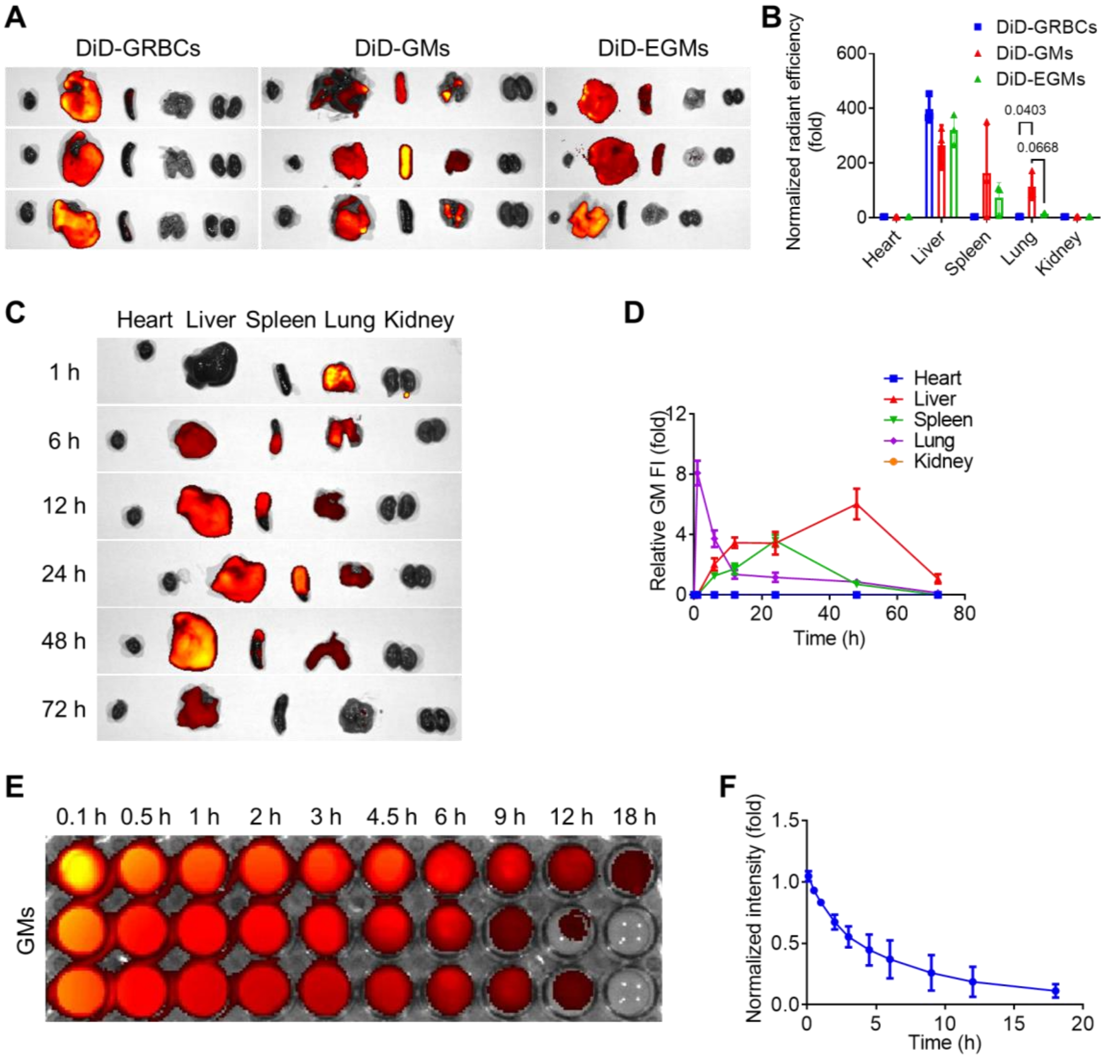

Supplementary Fig. 9. Ex vivo biodistribution and in vivo pharmacokinetics of GMs in AP mice. (**A** and **B**) AP mice (n=3) were *i.v.* administered with DiD-GMs, DiD-EGMs and DiD-GRBCs at the same fluorescence intensity, which referred to 5x10^6 of DiD stained GMs and EGMs, and 2x10^7 of DiD stained GRBCs, respectively. After injection for 6 h, different organs (heart, liver, spleen, lung and kidney) were collected for ex vivo fluorescence imaging (A), and the fluorescence intensity was quantified by IVIS (B). (**C** and **D**) AP mice were *i.v.* injected with 5x10^6 of Red CFDA-labelled GMs (n=3). The organs (heart, liver, spleen, lungs and kidneys) were collected for ex vivo fluorescent imaging after administration for different durations (1, 6, 12, 24, 48 and 72 h) (C), and the change of fluorescence intensity was quantified by IVIS (D). (**E** and **F**) Blood was collected from the other batch of mice (n=3) for ex vivo fluorescent imaging at pre-determined timepoints (0.1, 0.5, 1, 2, 3, 4.5, 6, 12, and 18 h) (E), and the change of fluorescence intensity was quantified by IVIS (F). All data was presented as mean ± s.d. (n = 3). All statistical analyses were conducted by using Two-Way ANOVA.

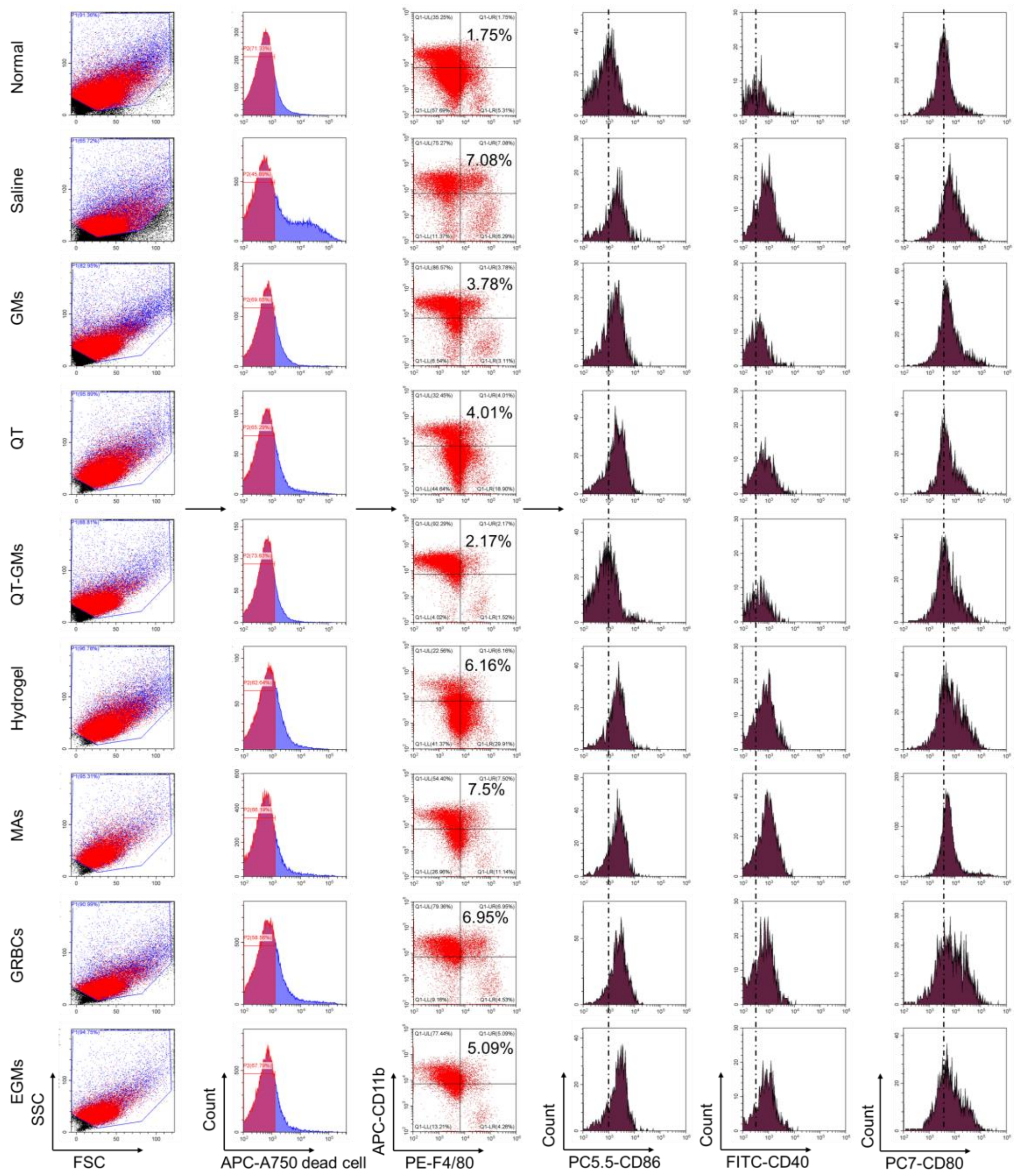

Supplementary Fig. 10. Gating strategy of flow cytometry analysis on the filtration of macrophages (F4/80$^+$CD11b$^+$ cells) and proinflammation polarized macrophages (the ratio of CD86$^+$ Cells, CD40$^+$ cells and CD80$^+$ cells in F4/80$^+$CD11b$^+$ cells) in lung tissues. AP mice were blindly and randomly divided into four groups (n=3), and i.v. administered with saline, QT, QT-GMs, GMs and EGMs at 3x10^6 of GMs per mouse, 5 mg kg^{-1} of QT, 2 mg kg^{-1} of hydrogel, 3x10^6 of MAs and 3x10^6 of GRBCs. After administration for 6 h, the lung tissues were collected for flow cytometry analysis.

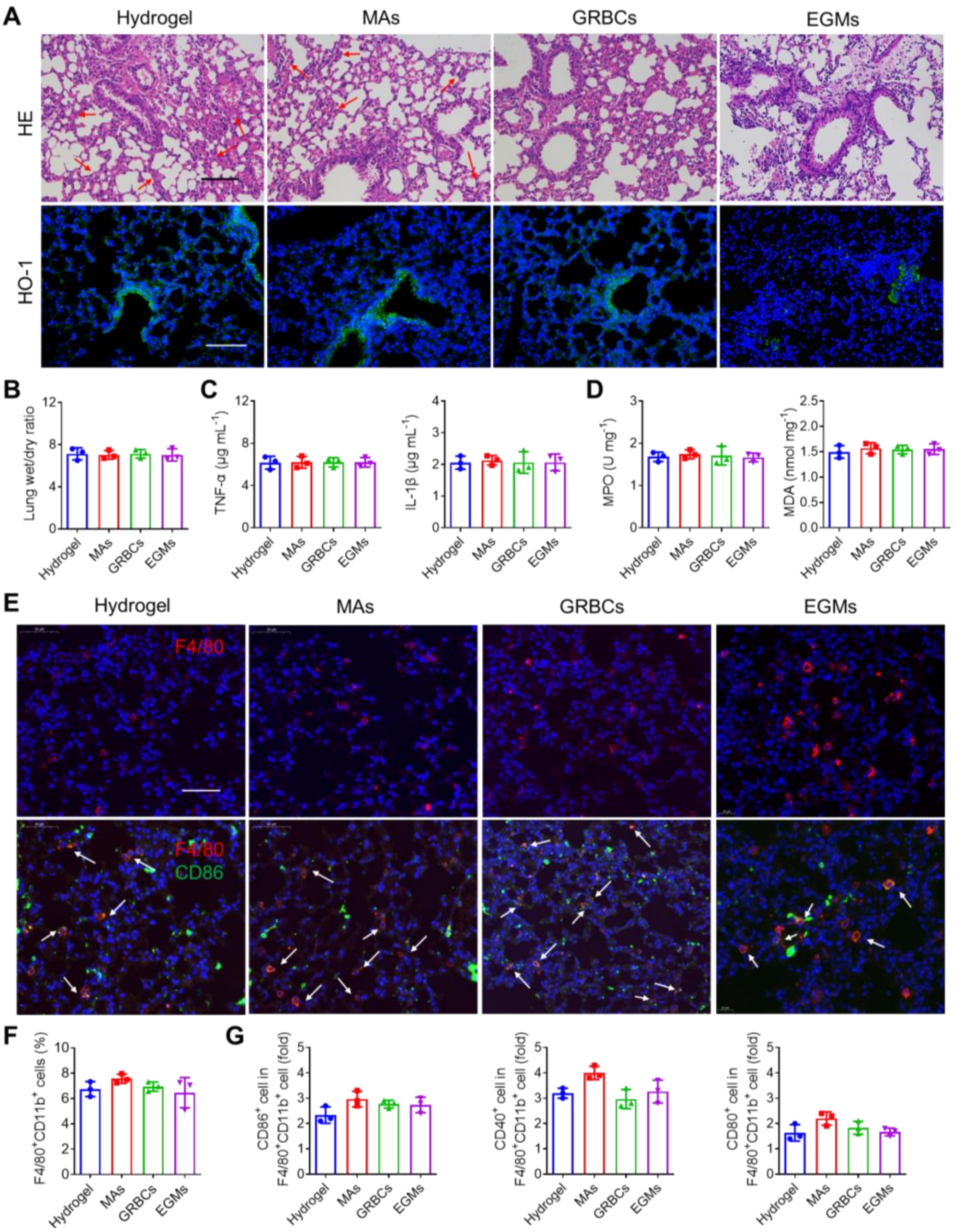

Supplementary Fig. 11. Therapeutic efficacy of hydrogel, MAs and GRBCs on acute lung inflammation in AP mice. (**A**) AP mice were i.v. administered with 2 mg kg^{-1} of hydrogel, and $3x10^6$ of MAs, EGMs and GRBCs, respectively. After administration for 6 h, the lung tissues were collected for HE staining and HO-1 staining. Scale bar: 100 μm. (**B**) The wet/dry ratio of the collected lung tissues. (**C**) Serum levels of TNF-α and IL-1β. (**D**) The concentrations of MPO and MDA in the lung tissues were analyzed by assay kits. (**E**) Fluorescence imaging on the filtration of $F4/80^+$ cells in the lungs of treated mice. Scale bar: 50 μm. (**F** and **G**) Flow cytometry analysis on the filtration of $F4/80^+CD11b^+$ cells and the ratio of $CD86^+$ cells, $CD40^+$ cells, $CD80^+$ cells among them in the lungs of treated mice. Representative photos in (A) and (E) came from independent experiment on three different lungs (n=3). All data was presented as mean ± s.d. (n = 3). All statistical analyses were conducted using One-Way ANOVA.

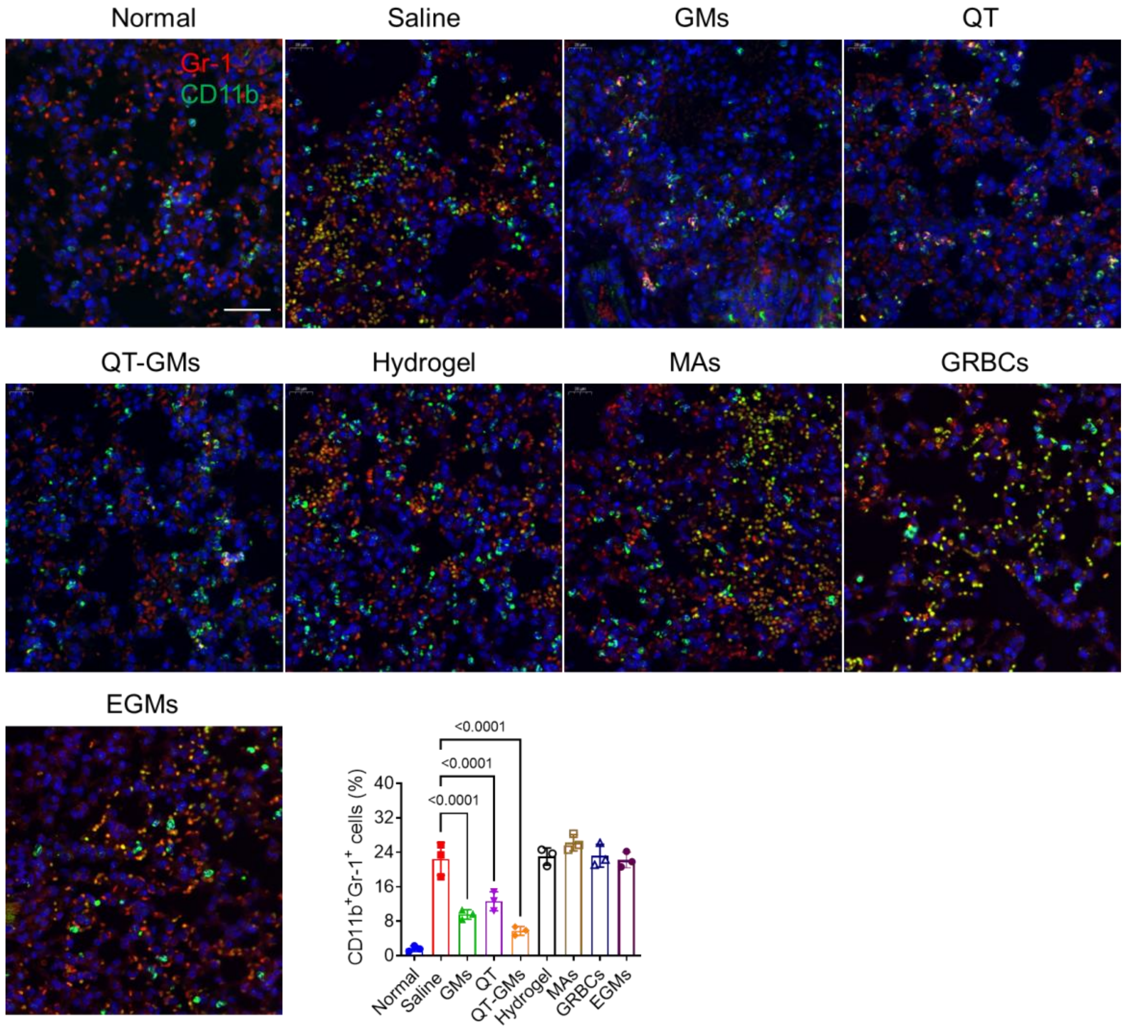

Supplementary Fig. 12. Fluorescence imaging on the filtration of neutrophils ($CD11b^{+}Gr\text{-}1^{+}$ cells) in the lungs of treated mice, and semi-quantitative analysis by ImageJ software. Scale bar: 40 μm. Representative photos came from independent experiments on three different lungs (n=3). All data was presented as mean ± s.d. All statistical analyses were conducted using One-Way ANOVA.

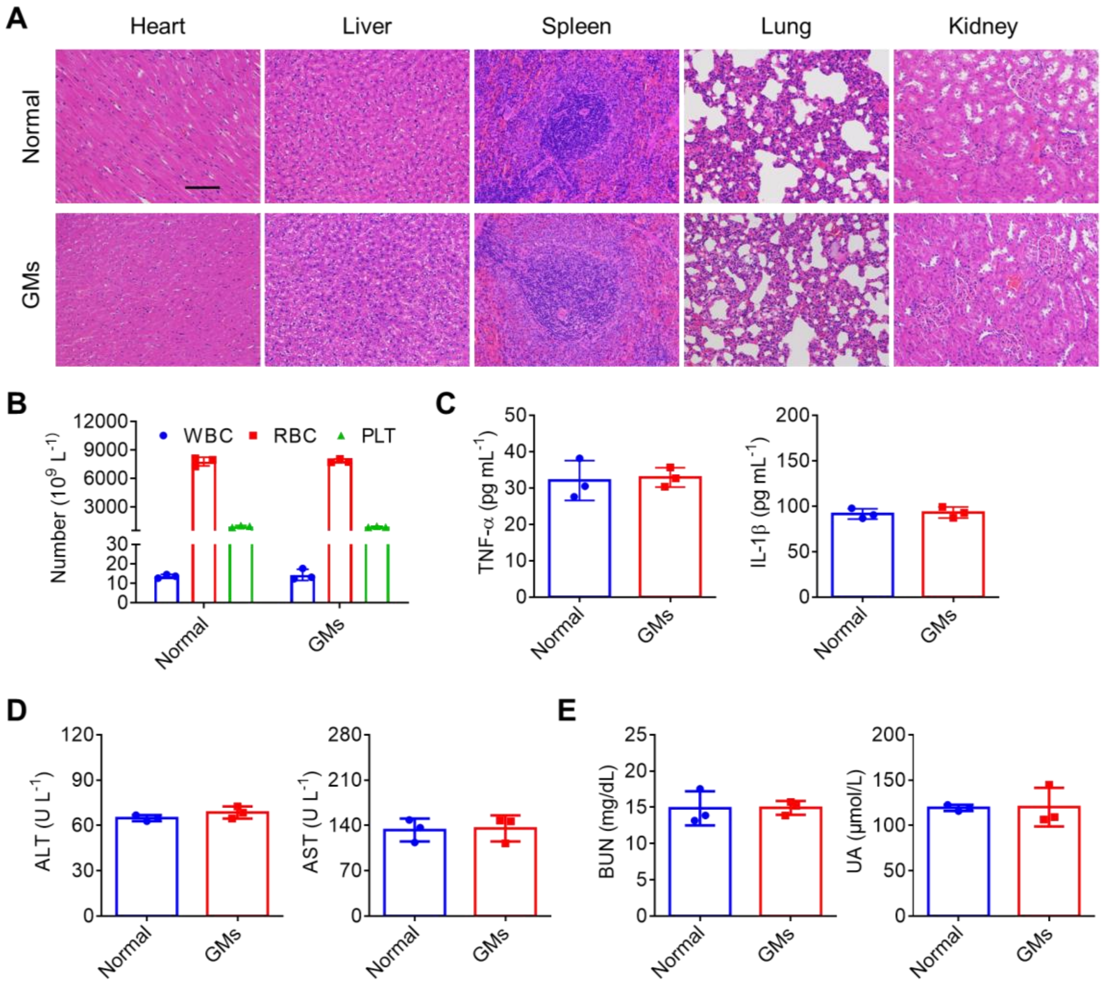

Supplementary Fig. 13. In vivo safety evaluation of GMs on SD rat. (**A**) The SD rats were i.v. administered with PBS and $5x10^6$ of GMs, respectively, once every four days with a total of 8 times. HE staining was conducted in the heart, liver, spleen, lung, and kidney. Scale bar: 100 µm. (**B**) The number of WBCs in blood was measured by hemocytometer. (**C**) The serum level of TNF-α and IL-1β. (**D** and **E**) The level of ALT, AST (D), BUN and UA (E) in the serum were analyzed. The experiments were repeated for three times (n = 3) and data was presented as mean ± s.d. All statistical analyses were conducted using One-Way ANOVA.

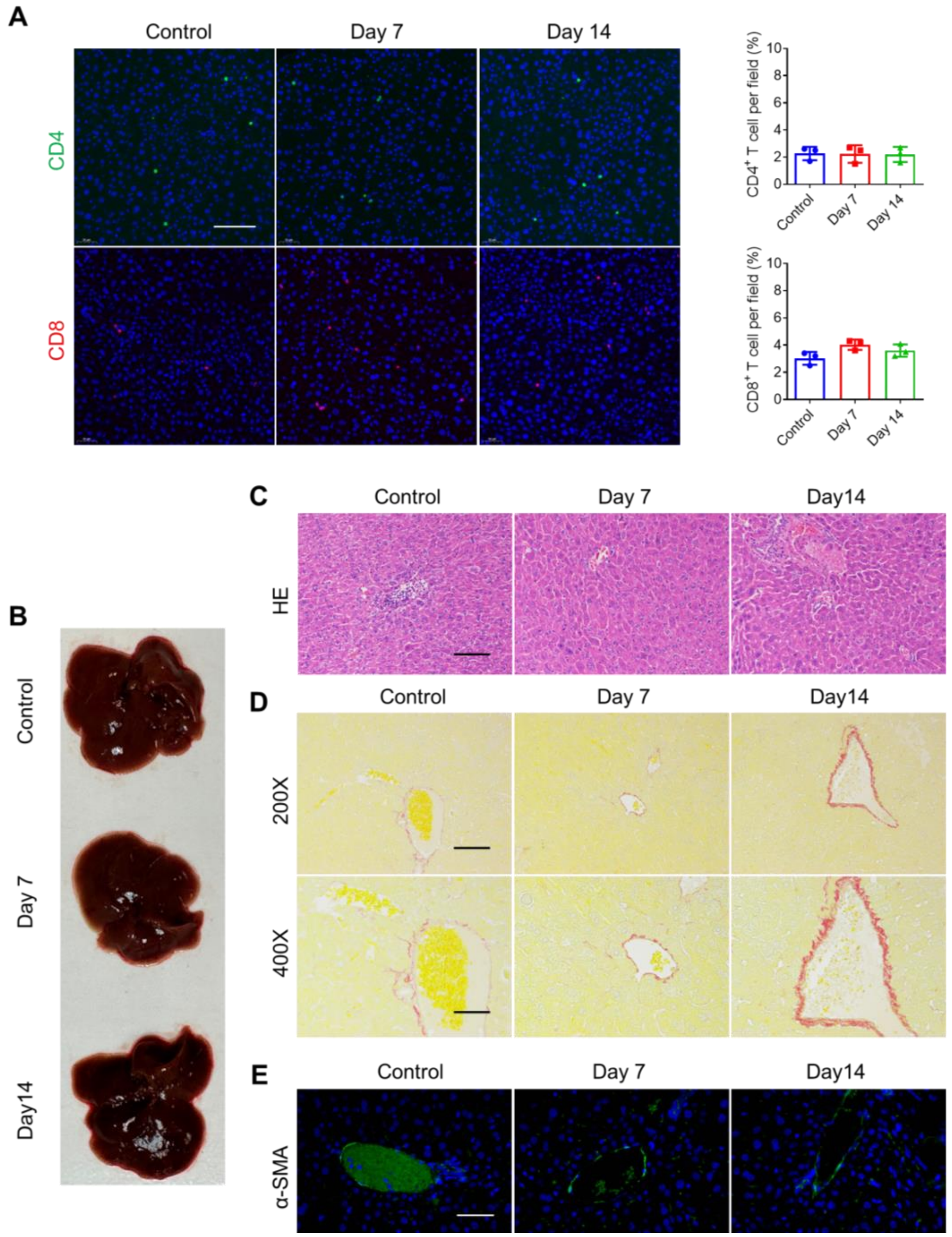

Supplementary Fig. 14. Evaluation of autoimmune response in liver from GMs treated mice. (**A**) C57BL/6 mice were i.v. administered with 5x10^{6} of DiD-GMs for different times (7 days and 14 days). Immunofluorescent staining of CD4 (blue) and CD8 (red) was conducted in liver sections. Scale bar: 100 μm. (**B**) Liver appearance after treatment for 7 days and 14 days. (**C**) HE staining on mouse liver sections after treatment for 7 days and 14 days. Scale bar: 100 μm. (**D**) Sirius red staining on mouse liver sections after treatment for 7 days and 14 days. Scale bar: 100 μm and 50 μm (×200 and ×400 magnification). (**E**) α-SMA staining on mouse liver sections after treatment for 7 days and 14 days. Scale bar: 50 μm. Representative photos in (A), (B), (C), (D) and (E) came from independent experiments on three different livers (n=3). All data was presented as mean ± s.d. All statistical analyses were conducted using One-Way ANOVA.

Supplementary Table 1. Dilutions and catalogue numbers of all used antibodies.

Antibody name	Category No.	Manufacturer	Dilution ratio
IL-6Rβ Monoclonal antibody	67766-1-Ig	Proteintech	1:1000
IL-6Rα Polyclonal antibody	23457-1-AP	Proteintech	1:500-
TNFR1 Polyclonal antibody	21574-1-AP	Proteintech	1:500
TNFR2 Polyclonal antibody	19272-1-AP	Proteintech	1:500
IL-1R2 Monoclonal antibody	60262-1-Ig	Proteintech	1:500
GAPDH (14C10) Rabbit mAb (Biotinylated) antibody	#5014	Cell signaling technology	1:1000
Recombinant Anti-Sodium Potassium ATPase antibody	ab76020	Abcam	1:100000
CoraLite488-conjugated Goat Anti-Rabbit IgG	SA00013-2	Proteintech	1:100
CoraLite594-conjugated Goat Anti-Mouse IgG	SA00013-3	Proteintech	1:100
TEM1 Monoclonal antibody	60170-1-Ig	Proteintech	1:100
HO-1/HMOX1 Polyclonal antibody	10701-1-AP	Proteintech	1:100
Anti-Mouse Ly-6G (Gr-1)	65140-1-Ig	Proteintech	N/A
PerCP/Cyanine5.5 anti-mouse CD86 Antibody	105027	Biolegend	1:20
APC anti-mouse CD206 (MMR) Antibody	141707	Biolegend	1:40
PE anti-mouse F4/80 Recombinant Antibody	157303	Biolegend	1:40
FITC anti-mouse/human CD11b Antibody	101205	Biolegend	1:200
PE anti-mouse I-Ab Antibody	116407	Biolegend	1:100
FITC anti-mouse CD40 Antibody	124607	Biolegend	1:50
PE/Cyanine7 anti-mouse CD80 Antibody	104733	Biolegend	1:40
