# Supplementary material for: Targeted therapies of inflammatory diseases with intracellularly gelated macrophages in mice and rats
Source: Nat Commun. 2024 Jan 6;15:328. doi: 10.1038/s41467-023-44662-5 (PMC10771422; doi:10.1038/s41467-023-44662-5)
Supplement: Supplementary file 3 — Reporting Summary [file 41467_2023_44662_MOESM3_ESM.pdf]

nature portfolio

Corresponding author(s): Ruibing Wang

Last updated by author(s): Dec 26, 2023

Reporting Summary

Nature Portfolio wishes to improve the reproducibility of the work that we publish. This form provides structure for consistency and transparency in reporting. For further information on Nature Portfolio policies, see our Editorial Policies and the Editorial Policy Checklist.

Statistics

For all statistical analyses, confirm that the following items are present in the figure legend, table legend, main text, or Methods section.

n/a	Confirmed	
☐	☒	The exact sample size (n) for each experimental group/condition, given as a discrete number and unit of measurement
☐	☒	A statement on whether measurements were taken from distinct samples or whether the same sample was measured repeatedly
☐	☒	The statistical test(s) used AND whether they are one- or two-sided<br>*Only common tests should be described solely by name; describe more complex techniques in the Methods section.*
☐	☒	A description of all covariates tested
☐	☒	A description of any assumptions or corrections, such as tests of normality and adjustment for multiple comparisons
☐	☒	A full description of the statistical parameters including central tendency (e.g. means) or other basic estimates (e.g. regression coefficient) AND variation (e.g. standard deviation) or associated estimates of uncertainty (e.g. confidence intervals)
☐	☒	For null hypothesis testing, the test statistic (e.g. F, t, r) with confidence intervals, effect sizes, degrees of freedom and P value noted<br>*Give P values as exact values whenever suitable.*
☒	☐	For Bayesian analysis, information on the choice of priors and Markov chain Monte Carlo settings
☒	☐	For hierarchical and complex designs, identification of the appropriate level for tests and full reporting of outcomes
☐	☒	Estimates of effect sizes (e.g. Cohen's d, Pearson's r), indicating how they were calculated

Our web collection on statistics for biologists contains articles on many of the points above.

Software and code

Policy information about availability of computer code

Data collection	SoftMax Pro 5.4.1 was utilized for plate based absorbance date collection. Zetasizer Software (version 7.11) was used for obtaining diameter and zeta potential. Gatan DiitalMicrograph3.9 was used for TEM analysis. Flow cytometry data was collected by using BD Accuri C6 Software (version 1.0.264.21). IVIS (Lumina XR III) was utilized for in vivo fluorescence imaging and LAS X (version 3.5.2) was used for in vitro fluorescence imaging.
Data analysis	FlowJo software (version 7.6.1) was used for flow cytometry analysis. Graphpad Prism 10 were used for statistical analysis and data plotting. ImageJ 1.8.0.345 was used to quantified fluorescent image.

For manuscripts utilizing custom algorithms or software that are central to the research but not yet described in published literature, software must be made available to editors and reviewers. We strongly encourage code deposition in a community repository (e.g. GitHub). See the Nature Portfolio guidelines for submitting code & software for further information.

Data

Policy information about availability of data

All manuscripts must include a data availability statement. This statement should provide the following information, where applicable:

- Accession codes, unique identifiers, or web links for publicly available datasets
- A description of any restrictions on data availability
- For clinical datasets or third party data, please ensure that the statement adheres to our policy

All data generated in this study are provided in the manuscript, Supplementary Information and Source Data file.

Research involving human participants, their data, or biological material

Policy information about studies with human participants or human data. See also policy information about sex, gender (identity/presentation), and sexual orientation and race, ethnicity and racism.

Reporting on sex and gender	N/A
Reporting on race, ethnicity, or other socially relevant groupings	N/A
Population characteristics	N/A
Recruitment	N/A
Ethics oversight	N/A

Note that full information on the approval of the study protocol must also be provided in the manuscript.

Field-specific reporting

Please select the one below that is the best fit for your research. If you are not sure, read the appropriate sections before making your selection.

☒ Life sciences ☐ Behavioural & social sciences ☐ Ecological, evolutionary & environmental sciences

For a reference copy of the document with all sections, see nature.com/documents/nr-reporting-summary-flat.pdf

Life sciences study design

All studies must disclose on these points even when the disclosure is negative.

Sample size	Sample size were estimated to achieve about 90% power for detection of significant differences between groups based on means and standard deviations. The source of each cell line was stated in Method section and figure legends.
Data exclusions	No date were excluded from the analysis.
Replication	All the experiments were repeated independently for three times. All attempts of replication were successful.
Randomization	The samples for in vitro and in vivo were randomly arranged into different experimental groups.
Blinding	The investigators were blinded to group arrangement during experiments, data collection and analysis.

Reporting for specific materials, systems and methods

We require information from authors about some types of materials, experimental systems and methods used in many studies. Here, indicate whether each material, system or method listed is relevant to your study. If you are not sure if a list item applies to your research, read the appropriate section before selecting a response.

Materials & experimental systems

n/a	Involved in the study	
	x	Antibodies
	x	Eukaryotic cell lines
x		Palaeontology and archaeology
	x	Animals and other organisms
x		Clinical data
x		Dual use research of concern
x		Plants

Methods

n/a	Involved in the study	
x		ChIP-seq
	x	Flow cytometry
x		MRI-based neuroimaging

Antibodies

Antibodies used

The following antibodies were used for WB analysis.
IL-6Rβ Monoclonal antibody (Cat No. 67766-1-Ig, Proteintech)
IL-6Rα Polyclonal antibody (Cat No. 23457-1-AP, Proteintech)
TNFR1 Polyclonal antibody (Cat No. 21574-1-AP, Proteintech)
TNFR2 Polyclonal antibody (Cat No. 19272-1-AP, Proteintech)
IL-1R2 Monoclonal antibody (Cat No. 60262-1-Ig, Proteintech)
GAPDH (14C10) Rabbit mAb (Biotinylated) antibody (Cat No. #5014, Cell signaling technology)
Recombinant Anti-Sodium Potassium ATPase antibody (Cat No. ab76020, Abcam)

The following antibodies were used for immunohistochemical and immunofluorescent staining.
CoraLite488-conjugated Goat Anti-Rabbit IgG (Cat No. SA00013-2, Proteintech)
CoraLite594-conjugated Goat Anti-Mouse IgG (Cat No. SA00013-3, Proteintech)
TEM1 Monoclonal antibody (Cat No. 60170-1-Ig, Proteintech)
HO-1/HMOX1 Polyclonal antibody (Cat No. 10701-1-AP, Proteintech)
Anti-Mouse Ly-6G (Gr-1) (Cat No. 65140-1-Ig, Proteintech)
Gp130 antibody (21175-1-AP, Proteintech)
CD248 antibody (Cat. No. 60170-1-1g, Proteintech)
Fibronectin antibody (Cat. No. 66042-1-1g, Proteintech)

The following antibodies were used for flow cytometry.
PerCP/Cyanine5.5 anti-mouse CD86 Antibody (Cat No. 105027, Biolegend)
APC anti-mouse CD206 (MMR) Antibody (Cat No. 141707, Biolegend)
PE anti-mouse F4/80 Recombinant Antibody (Cat No. 157303, Biolegend)
FITC anti-mouse/human CD11b Antibody (Cat No. 101205, Biolegend),
PE anti-mouse I-Ab Antibody (Cat No. 116407, Biolegen)
FITC anti-mouse CD40 Antibody (Cat No. 124607, Biolegen)
PE/Cyanine7 anti-mouse CD80 Antibody (at No. 104733, Biolegen)

Validation

The validation and application of antibodies were described in manufacture's website.
IL-6Rβ Monoclonal antibody (Cat No. 67766-1-Ig, Proteintech), Reactivity: Human, Mouse, validated by SDS-PAGE and western blot.
IL-6Rα Polyclonal antibody (Cat No. 23457-1-AP, Proteintech), Reactivity: Human, Mouse, Rat, validated by SDS-PAGE and western blot.
TNFR1 Polyclonal antibody (Cat No. 21574-1-AP, Proteintech), Reactivity: Human, Mouse, validated by SDS-PAGE and western blot.
TNFR2 Polyclonal antibody (Cat No. 19272-1-AP, Proteintech), Reactivity: Human, Mouse, validated by SDS-PAGE and western blot.
IL-1R2 Monoclonal antibody (Cat No. 60262-1-Ig, Proteintech), Reactivity: Human, Mouse, validated by SDS-PAGE and western blot.
GAPDH (14C10) Rabbit mAb (Biotinylated) antibody (Cat No. #5014, Cell signaling technology), Reactivity: Human, Mouse, Rat, Rabbit, validated by alidated by SDS-PAGE and western blot.
Recombinant Anti-Sodium Potassium ATPase antibody (Cat No. ab76020, Abcam), Reactivity: Mouse, Rat, Human, validated by SDSPAGE
and western blot.
CoraLite488-conjugated Goat Anti-Rabbit IgG (Cat No. SA00013-2, Proteintech), Reactivity: Rabbit, validated by immunofluorescent anlaysis.
CoraLite594-conjugated Goat Anti-Mouse IgG (Cat No. SA00013-3, Proteintech), Reactivity: Mouse, validated by immunofluorescent anlaysis.
TEM1 Monoclonal antibody (Cat No. 60170-1-Ig, Proteintech), Reactivity: Human, Mouse, validated by SDS-PAGE and western blot.
HO-1/HMOX1 Polyclonal antibody (Cat No. 10701-1-AP, Proteintech), Reactivity: Human, Mouse, Rat, validated by SDS-PAGE and western blot.
Anti-Mouse Ly-6G (Gr-1) (Cat No. 65140-1-Ig, Proteintech), Reactivity: Mouse, validated by flow cytometry.
Gp130 antibody (21175-1-AP, Proteintech), Reactivity: Mouse, validated by immunofluorescent analysis, western blot and IHC staining.
CD248 antibody (Cat. No. 60170-1-1g, Proteintech), Reactivity: Mouse, validated by western blot and IHC staining.
Fibronectin antibody (Cat. No. 66042-1-1g, Proteintech), Reactivity: Human, Mouse and Rat, validated by western blot, IHC staining, IP experiment and IF staining.

PerCP/Cyanine5.5 anti-mouse CD86 Antibody (Cat No. 105027, Biolegend), Reactivity, Mouse, validated by flow cytometry.
APC anti-mouse CD206 (MMR) Antibody (Cat No. 141707, Biolegend), Reactivity, Mouse, validated by flow cytometry.
PE anti-mouse F4/80 Recombinant Antibody (Cat No. 157303, Biolegend), Reactivity, Mouse, validated by flow cytometry.
FITC anti-mouse/human CD11b Antibody (Cat No. 101205, Biolegend), Reactivity, Human, Mouse, validated by flow cytometry.

Eukaryotic cell lines

Policy information about cell lines and Sex and Gender in Research

Cell line source(s)	Mouse macrophage cell line (RAW264.7 cell), human umbilical vein endothelial cell (HUVEC), and rat synovial cell (RSC-364 cell) were purchased from ATCC (USA).
Authentication	These cells were authenticated by mycoplasma detection, isozyme detection, and DNA fingerprinting.
Mycoplasma contamination	These cells were detected for mycoplasma contamination and no mycoplasma was found.
Commonly misidentified lines (See ICLAC register)	No commonly misidentified lines were used.

Animals and other research organisms

Policy information about studies involving animals; ARRIVE guidelines recommended for reporting animal research, and Sex and Gender in Research

Laboratory animals	6-week-old female SD rats and 6-week-old C57BL/6 male mice used in this study were maintained in a dedicated pathogen-free animal facility at 60 % of humidity and 25 °C with 12/12 light schedule, and free access to food and water in the Shenzhen Institute of Translational Medicine.
Wild animals	This study did not involve wild animals.
Reporting on sex	6-week-old female SD rats and 6-week-old C57BL/6 male mice used in this study.
Field-collected samples	This study did not involved field-collected samples.
Ethics oversight	All animal experiments were approved by the Animal Ethics Committee, China Technology Industry Holdings (Shenzhen) Co., Ltd, and were conducted in accordance with the Animal Management Rules of the Ministry of Health of the P. R. China.

Note that full information on the approval of the study protocol must also be provided in the manuscript.

Plants

Seed stocks	N/A
Novel plant genotypes	N/A
Authentication	N/A

Flow Cytometry

Plots

Confirm that:

- [x] The axis labels state the marker and fluorochrome used (e.g. CD4-FITC).
- [x] The axis scales are clearly visible. Include numbers along axes only for bottom left plot of group (a 'group' is an analysis of identical markers).
- [x] All plots are contour plots with outliers or pseudocolor plots.
- [x] A numerical value for number of cells or percentage (with statistics) is provided.

Methodology

Sample preparation	Cells were detached from flasks and immediately washed with PBS before flow cytometry analsyis.

Instrument	BD Acurri C6 cytometer
Software	BD accuri C6 software (version 1.0.264.21) and Flowjo sofeware (version 7.6) were used for flow cytometry analysis.
Cell population abundance	No post-sort fractions were collected. Flow cytometry was used for quantitative analysis only.
Gating strategy	Initial cell populatons were gated for a live population using FSC and SCC plot of cell only sample The gate was set to remove cell debris and dead cells.

☒ Tick this box to confirm that a figure exemplifying the gating strategy is provided in the Supplementary Information.
